# Role of hepatitis B surface antigen in the development of hepatocellular carcinoma: regulation of lymphoid enhancer-binding factor 1

**DOI:** 10.1186/1756-9966-28-58

**Published:** 2009-04-29

**Authors:** Xiaochen Tian, Jinjun Li, Zhang-Mei Ma, Chao Zhao, Da-Fang Wan, Yu-Mei Wen

**Affiliations:** 1 Key Laboratory of Medical Molecular Virology, Shanghai Medical College, Fudan University, Shanghai, PR China; 2 Shanghai Cancer Institute, Shanghai, PR China; 3 Institutes of Biomedical Sciences, Fudan University, Shanghai, PR China

## Abstract

**Background:**

There are around 350 million of hepatitis B surface antigen (HBsAg) carriers worldwide, and among them, high risk of developing hepatocellular carcinoma (HCC) has been identified by epidemiological studies. To date, the molecular role of HBsAg in HCC development has not been fully studied. We have previously reported that in cell cultures, HBsAg up-regulated the expression of lymphoid enhancer-binding factor 1 (LEF-1), a key component of the *Wnt *pathway. In this study we aimed to study this effect of HBsAg on LEF-1 in the development of HCC.

**Methods:**

Expression of HBsAg, LEF-1 and its downstream effector genes were compared among 30 HCCs, their peritumor tissue counterparts and 9 normal control liver tissues by quantitative real-time PCR. In addition, immunohistochemical staining studies on HBsAg and LEF-1 expression were conducted among these samples.

**Results:**

The expression of LEF-1 was compared between 13 HBsAg positive HCC tissues and 17 HBsAg negative HCC tissues. Simultaneous detection of LEF-1 and HBsAg was observed in HBsAg positive HCC tissues and, additionally, the simultaneous detection of HBsAg and LEF-1 was more pronounced in peritumor tissues, compared to that in the tumor tissues. The distribution of cellular LEF-1 in peritumor tissues was predominantly in the cytoplasm; while LEF-1 in the tumor tissues was located either exclusively in the nucleus or both in the nucleus and cytoplasm. By real-time PCR, the expression levels of LEF-1 downstream effector genes *cyclin D1 *and *c-myc *were higher in peritumor cells compared to that of the tumor cells. However, a 38 kDa truncated isoform of LEF-1, rather than the 55 kDa wild-type LEF-1, was significantly elevated in the HBsAg positive tumor cells.

**Conclusion:**

Data indicate that deregulation of the *Wnt *pathway by HBsAg occurred in HBV-associated HCCs, but was more pronounced in the peritumor cells. It is speculated that HBsAg could stimulate proliferation and functional modification of hepatocytes via LEF-1 through the *Wnt *pathway at the pre-malignant stage.

## Background

Hepatitis B virus (HBV) is the prototype of *hepadnaviridae*. It is estimated that around 350 million people are carriers of hepatitis B surface antigen (HBsAg) worldwide [1,2]. Persistent HBV infection leads to chronic hepatitis, and is closely associated with the development of liver cirrhosis and hepatocellular carcinoma (HCC) [3]. Three forms of viral particles can be detected in the serum of HBV infected patients, namely, 42 nm diameter mature virion particles, 22 nm diameter spherical particles and 22 nm diameter filamentous particles [4]. Uniquely, 22 nm subviral particles, which are composed of HBsAg and do not contain viral DNA, usually outnumber the virions in patient serum by a factor of 1000-fold or more [5]. Though HBsAg has been identified as the neutralizing antigen of HBV and has been used as the major component of preventive vaccine for viral hepatitis B, persistence of HBsAg in serum of patients has been recognized as a high risk factor for development of HCC [6,7]. The possible roles of HBV envelope proteins LHBs (Pre-S1/Pre-S2/S) and MHBs (Pre-S2/S) in HCC development have been reported [8,9]. However, the role of major HBsAg in tumorigenesis has not been studied in detail.

By microarray study of cells transfected with the S gene coding for HBsAg, we have previously shown that marked up-regulation of lymphoid enhancer-binding factor 1 (LEF-1), a transcriptional factor in *Wnt *pathway, was closely correlated with HBsAg expression [10]. Furthermore, the expression level and cellular distribution of LEF-1 protein, mainly the dominant negative truncated isoform, was changed by the expression of HBsAg. In this study, we aimed to investigate the roles played by HBsAg on LEF-1 expression in the development of HBV-associated HCC. By immunohistochemical analysis and molecular studies, the intracellular expression and distribution of LEF-1 and HBsAg, *cyclin D1 *and *c-myc *gene expression were compared between HBsAg positive and negative HCC tissues, peritumor tissues and normal liver tissues. The possible roles of HBsAg in HCC development are discussed.

## Methods

### Human liver tissues

Thirty surgical resected HCC tissues from different individuals were provided by Shanghai Cancer Institute. Tissue samples were categorized as tumorous (T) or matched adjacent peritumorous liver tissues (pT) by hematoxylin and eosin (HE) stained sections under the microscope. The size and regions of the resection of the tumorous and peritumorous tissues were decided by the surgeons based on each individual case under the regulation of the ethics committee. All these HCCs were associated with HBV infection as defined by serum HBsAg positive. Normal liver tissues (NL) from liver transplantation donors (n = 9) were obtained from Shanghai Cancer Institute and First Affiliated Hospital, Zhejiang University School of Medicine (kindly provided by Dr. Shusen Zheng). All samples collected followed the regulations of the ethics committees of both hospitals.

### Immunohistochemical staining

Resected liver tissue samples were immediately immersed in 4% formalin and fixed for 18 to 24 h and paraffin-embedded. Immunohistochemical staining was carried out on tissue sections by using anti-LEF-1 polyclonal rabbit antibody (1:50, Abcam, Cambridge, UK) or anti-HBsAg monoclonal antibody (1:50, Changdao Biotech, Shanghai, China) to detect the expression of LEF-1 and HBsAg respectively.

### Reverse transcription and real-time PCR

After treated with 10 U DNase I (TaKaRa, Dalian, China) at 37°C for 30 min, 2 μg total RNA was reverse transcribed into cDNA by SuperScript II reverse transcriptase (Invitrogen, Carisbad CA, USA) according to the manufacturer's protocol. Quantitative real-time PCR was carried out using specific primer pairs designed by PrimerBank [11]. For real-time PCR, 2 μl of 10-fold dilutions of the cDNA products were assayed using the Premix Ex Taq Perfect Real Time PCR kit (TaKaRa, Dalian, China). To assess the association of HBsAg and LEF-1 isoforms in HCC tissues, two pairs of primers were designed to detect different LEF-1 isoforms. Primers LP1 and LP2 were designed to target the β-catenin binding domain, which could differentiate the 38 kDa truncated LEF-1 isoform from the 55 kDa full-length LEF-1 [12]. Another pair of primers LP3 and LP4 was targeted to the 3' UTR region of LEF-1 mRNA, and thus could detect both the full length and the isoforms. The house keeping gene GAPDH was used as an internal control. All experiments were performed twice independently. Primers used in this study are listed in Table 1.

**Table 1:** Primers of real-time PCR in this study

Gene	Primers
GAPDH	Forward: GGTATCGTGGAAGGACTCATGAC
	Reverse: ATGCCAGTGAGCTTCCCGTTCAG
LEF-1-full length (LP1 and LP2)	Forward: AATCATCCCGGCCAGCA
	Reverse: TGTCGTGGTAGGGCTCCTC
LEF-1-isoform (LP3 and LP4)	Forward: CATAGTGGCTTCTCCGCCCTTGTAAG
	Reverse: TTCAAGTGCTGGGCTTTTTACAACAAG
*cyclin D1*	Forward: GCTGGAGCCCGTGAAAAAGA
	Reverse: CTCCGCCTCTGGCATTTTG
*c-myc*	Forward: CTGTATGTGGACGGCTTCTCG
	Reverse: CTGCTGTCGTTGAGAGGGTAG

### Statistical analysis

The Wilcoxon signed rank tests were performed to evaluate the difference of expression levels of LEF-1, *cyclin D1 *and *c-myc *between HCC tissues and normal tissues. Tests were considered of statistical significance when their *p *values were less than 0.05.

## Results

### Expression and distribution of HBsAg and LEF-1 protein in HCC tissues

**Figure 1:**
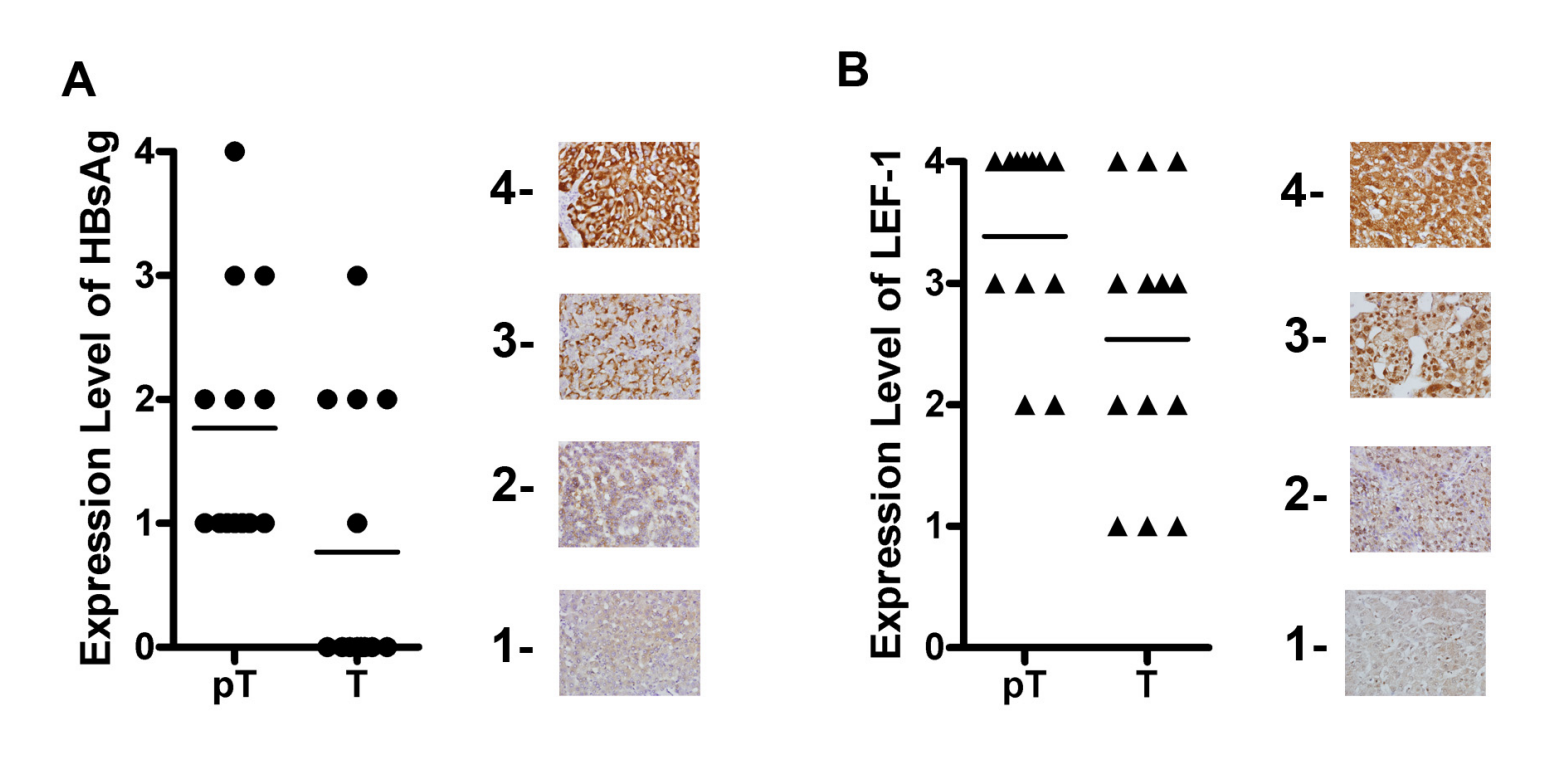
**Correlation between HBsAg and LEF-1 expression levels in HCC tissues**. Expression levels of HBsAg (A) and LEF-1 (B) were analyzed by the immunohistochemical studies in 13 HBsAg positive HCC tissues. LEF-1 expression was positively correlated with HBsAg expression. The units of expression levels were set arbitrarily which were defined according to the color density by immunohistochemical staining. The examples of arbitrary units of color density are shown (1 faint brown, 2 median brown, 3 brown, 4 dark brown).

**Figure 2:**
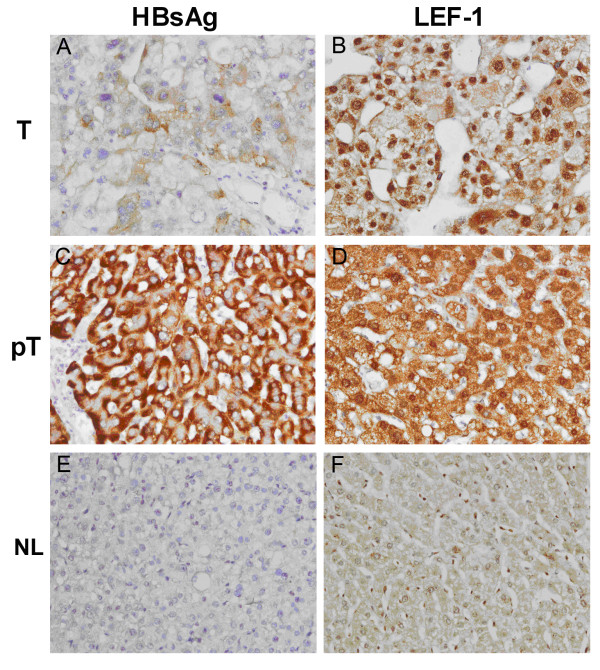
**Intracellular expression and distribution of HBsAg and LEF-1 in liver tissue sections**. HBsAg (A, C, E) and LEF-1 protein (B, D, F) expression in liver tissues sections was determined by immunohistochemical staining using anti-HBsAg monoclonal antibody and anti-LEF-1 rabbit polyclonal antibody respectively (400× magnification). HBsAg and LEF-1 expression and cellular distribution were studied and compared in tumor tissues (T) (A, B), peritumor tissues (pT) (C, D) and normal liver tissues (NL) (E, F). As shown, HBsAg was expressed at lower level in tumor tissues compared to that of peritumor tissues, and LEF-1 was found exclusively in the nucleus in tumor tissues, whereas it was mainly detected in the cytoplasm in peritumor tissues.

Immunohistochemical staining of the HCC tissues showed that HBsAg was detected in 13 of 30 HCC tissues, either in tumor cells or peritumor cells. HBsAg was detected only in 5 out of the 13 tumor tissues, while in the paired peritumor tissues, HBsAg was observed in all 13 samples (Table 2). LEF-1 was detected in both tumor cells and peritumor cells of all 30 HCC tissues, with no significant difference between tumor cells and peritumor cells. When LEF-1 expression level was analyzed in the HBsAg positive tissues, it was simultaneously associated with the expression levels of HBsAg (Figure 1 and Table 2). The exspression of LEF-1 was found more pronounced in peritumor tissues, compared to that in the tumor tissues among HBsAg positive HCC samples, whereas, no significant differences of LEF-1 expression were observed between tumor cells and peritumor cells in the other 17 HBsAg negative tissues. Cellular distribution pattern of LEF-1 protein was compared between peritumor cells and tumor cells of HBsAg positive tissues. LEF-1 protein was located either exclusively in the nucleus or both in the nucleus and cytoplasm of tumor cells, whereas in peritumor cells LEF-1 was located predominantly in the cytoplasm (Figure 2 and Table 2). When the expression of LEF-1 protein was compared with that of HBV negative normal liver tissues, marked up-regulation of LEF-1 was observed both in tumor tissues and the peri-tumor tisseus among all of 30 HCC tissues. The cellular location of LEF-1 in normal liver cells was in the cytoplasm, more closely representing that in peritumor cells (Figure 2).

**Table 2:** The expression pattern and intracellular distribution of HBsAg and LEF-1 in 13 HBsAg positive HCC tissues.

		Peritumor Tissue (%)	Tumor Tissue (%)	P value
HBsAg expression		13/13 (100)	5/13 (38.5)	
LEF-1 intracelluler location	Nucleus	4/13 (30.8)	9/13 (69.2)	
	Cytoplasm	7/13 (53.8)	0/13 (0)	
	Cytoplasm & Nucleus	2/13 (15.3)	4/13 (30.8)	
LEF-1 isoforms abundance*	38 kDa LEF-1	2.69 ± 2.26E-03	2.34 ± 3.64E-02	0.03
	55 kDa LEF-1	1.49 ± 2.30E-02	1.51 ± 1.90E-02	0.98

* Results are the arbitary units which represent the relative abundance of LEF-1 mRNA.

### Deregulation of LEF-1 isoforms in HCC tissues

**Figure 3:**
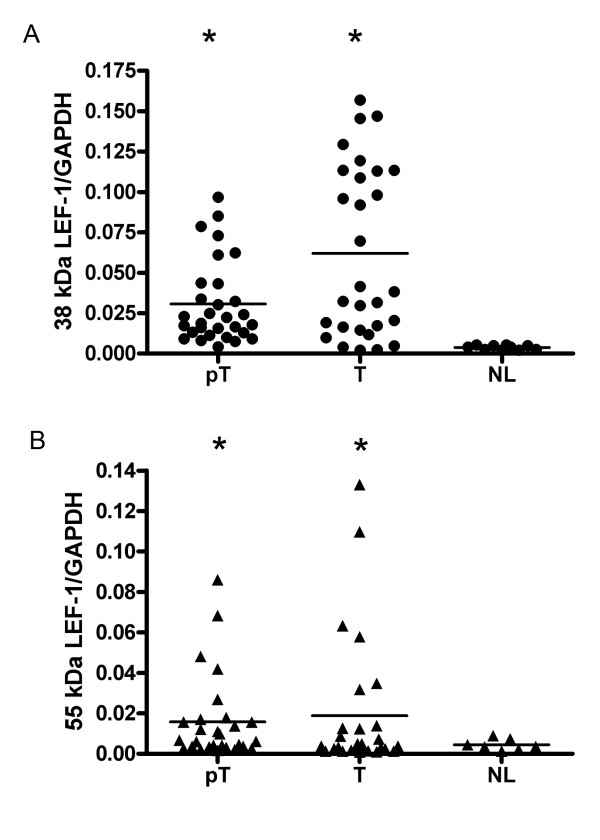
**Expression levels of LEF-1 isoforms in HCC tissues**. By real-time PCR, the expression levels of 38 kDa truncated isoform of LEF-1 (A) and 55 kDa full-length LEF-1 (B) were compared in tumor tissues (T), peritumor tissues (pT) and normal liver tissues (NL). The value of the Y axis is the arbitrary unit which reflects the relative abundance of LEF-1. The GAPDH was used as an internal control of real-time PCR. The expression levels of LEF-1 isoforms were significantly induced in tumor tissues compared to that of peritumor tissues and normal liver tissues (* p < 0.05).

The expression pattern of LEF-1 isoforms was studied in HCC tissues by quantitative real-time PCR. Results showed that compared to that of normal liver tissues by real-time PCR, both 38 kDa truncated isoform and 55 kDa full-length LEF-1 were markedly increased in tumor cells and peritumor cells (Figure 3). However, when compared to that in the peritumor cells, the 38 kDa truncated isoform of LEF-1 was more markedly induced in tumor cells, (Figure 3A), while the 55 kDa full-length LEF-1 did not show significant changes (Figure 3B). To further investigate the association of the expression pattern of LEF-1 isoforms and HBsAg expression, LEF-1 isoforms were analyzed in 13 HBsAg positive HCC tissues. The 38 kDa truncated isoform of LEF-1 was significantly up-regulated in tumor cells compared to that in the peritumor cells, while the 55 kDa full-length LEF-1 did not exhibit changes between tumor and peritumor cells (Table 2). However in the other 17 HBsAg negative HCC tissues, no significant changes were observed in either isoforms.

### Up-regulation of downstream target genes of Wnt pathway

**Figure 4:**
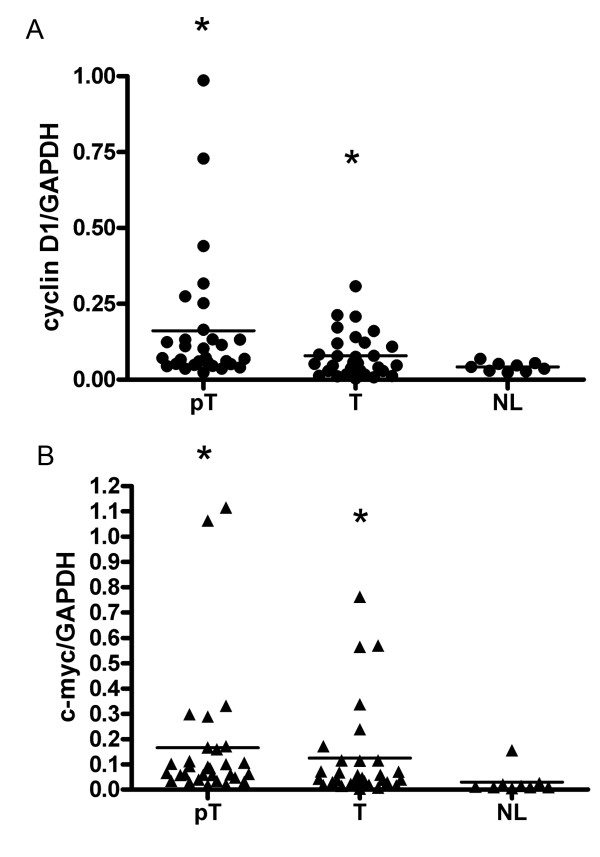
**Expression levels of *cyclin D1 *and *c-myc *in HCC tissues**. By real-time PCR, the expression levels of LEF-1 downstream effector genes *cyclin D1 *(A) and *c-myc *(B) were compared in tumor tissues (T), peritumor tissues (pT) and normal liver tissues (NL). The expression levels of *cyclin D1 *and *c-myc *were significantly induced in tumor tissues compared to that of peritumor tissues and normal liver tissues (* p < 0.05).

To further study the deregulation of *Wnt *pathway induced by aberrant up-regulation of LEF-1, expression levels of *c-myc *and *cyclin D1 *in HCC tissues and normal liver tissues were compared by real-time PCR. Results showed that compared to that of normal livers, the expression of *cyclin D1 *and *c-myc *was increased significantly in both tumor cells and peritumor cells of HCC tissues (Figure 4). In addition, the expression level of *cyclin D1 *was much higher in peritumor cells compared to that of tumor cells, and *c-myc *expression showed a similar pattern (Figure 4).

## Discussion

Hepatocellular carcinoma is the fifth most common malignancy worldwide [13]. Its risk factors include chronic infections by hepatitis B and C virus (HBV and HCV), and nonviral liver diseases [14,15]. Epidemiological study indicated that long term persistence of HBsAg in chronic hepatitis B patients is a risk factor for the development of HCC [7]. Extensive studies have been carried out to reveal the roles of HBV in contributing to proliferation and anti-apoptotic behavior of HCC cells [16,17]. Cumulative data suggested that HBx is a multifunctional regulatory viral protein, which interferes directly or indirectly with a variety of cellular functions including cell cycle progression, transformation and apoptosis [18-20]. Other groups reported that LHBs and MHBs functioned as trans-activators which induced cell proliferation and/or cell death of hepatocytes [21-23]. In this study we investigated the possible roles played by major HBs in tumorgenesis, and the association between HBsAg expression and *Wnt *signaling pathway deregulation in HBV-associated HCC tissues.

To reveal the implications of *in vivo *association between HBsAg and LEF-1 up-regulation in HCC, the expression levels of these two proteins were compared both by immunohistochemical staining and by real-time PCR among HCC tumor tissues, peritumor tissues and normal liver tissues. Experimental data have shown that the aberrant regulation of the canonical *Wnt *pathway was one of the important events involved in HCC development [24,25]. However, mutations in β-catenin or adenomatous polyposis coli (APC) genes, which appeared in over 90% of colorectal cancers [26,27] were found only in about 20–30% of HCCs [28], suggesting that the predominant mechanisms activating *Wnt *signaling pathway in HCCs could be different from that in other cancers. Bengochea et al reported that deregulation of Wnt/Frizzled receptor elements was common in human hepatocellular carcinoma [29], and disturbance of regulatory mechanisms other than mutations involving β-catenin is more likely of importance in HCC. Our results on the molecular expression levels among 30 HCC tissue and 9 normal liver tissues showed that HBsAg up-regulated not only LEF-1 but also the two of LEF-1 downstream genes expression levels. These finding provided evidence that HBsAg affected the *Wnt *pathway via up-regulation of LEF-1.

In this study, though all 30 HCC samples were collected from serum HBsAg positive patients, only 13 liver tissues were HBsAg positive by immunohistochemical staining. Since the expression pattern of LEF-1 was not significnatly changed in HBsAg negative HCC tissues, to reveal the roles of HBsAg on HCC development, we concentrated on these 13 pairs of HBsAg positive samples. Specifically, the expression levels of HBsAg, LEF-1, *cyclin D1 *and *c-myc *were studied in tumor cells and peritumor cells from the same patient. LEF-1 expression levels were found associated with the levels of HBsAg expression in HCC tissues. Interestingly, the intracellular distribution of LEF-1 protein in tumor cells was different from that in peritumor cells. In the peritumor cells LEF-1 was predominantly located in the cytoplasm, while in the tumor cells LEF-1 was located exclusively in the nucleus or both in the nucleus and cytoplasm. This observation is in accordance with a recent report stating that LEF-1/TCF was up-regulated in 52% of HCCs by strong nuclear LEF-1/TCF staining [30]. As we have previously observed that expression of HBsAg initiated transfer of LEF-1 from the cytoplasm into the nucleus, in this study, we further identified that the transfer of LEF-1 into the nucleus also occurred in tumor cells. The different distribution of LEF-1 in tumor cells and peritumor cells suggests that different mechanisms could be involved in the pre-malignant stage and the malignant stage in HBV associated HCC.

Our previous study showed that the 38 kDa truncated isoform of LEF-1 was markedly induced in HBsAg expressing cells, while full-length LEF-1 did not show a significant change. It was reported that the 55 kDa full-length LEF-1 contains three functional domains, namely, β-catenin binding domain, context-dependent activation domain (CAD) and HMG DNA binding domain, while the 38 kDa truncated isoform of LEF-1 which lacks the β-catanin binding domain derived from an intronic promoter and exhibits dominant negative activity [31,32]. To further investigate the expression of LEF-1 isoforms in HCCs, quantitative real-time PCR was employed to analyze the expression patterns of LEF-1 isoforms in 30 pairs of HCC tissues in tumor cells and peritumor cells. Compared to those in normal liver tissues, though both isoforms were significantly up-regulated in HCC, the 38 kDa truncated isoform of LEF-1 was more significantly up-regulated in tumor cells, than that in peritumor cells especially in those 13 HBsAg positive HCC tissues. The 55 kDa full-length LEF-1 showed no changes between tumor cells and peritumor cells. This observation further suggested that the molecular signaling cascades could have been changed between peritumor cells and tumor cells.

To further confirm the association of LEF-1 and HBsAg, expression pattern of LEF-1 in 13 HBsAg positive HCC tissues was analyzed and compared to that in 17 HBsAg negative HCC tissues. The expression of LEF-1 was found closely associated with the HBsAg expression in HBsAg positive HCC tissues. However no significant differences were observed either in LEF-1 protein or LEF-1 isoforms when compared between tumor cells and peritumor cells in these HBsAg negative tissues. The different expression patterns of LEF-1 between HBsAg positive and negative HCC tissues suggested that HBsAg could play important roles in regulating *Wnt *signaling pathway, thus providing new insights into the involvement of HBsAg in hepatocarcinogenesis. However, the molecular mechanisms of HBsAg-LEF-1 interaction and their roles in the development of HCC merit further investigation. Other viral or cellular factors might also be involved in the interaction between HBV and *Wnt *pathway. For instance, HBx has been reported to be essential for the activation of Wnt/b-catenin signalling in hepatoma cells [33], and reduced the phosphorylation level of b-catenin by suppressing GSK-3b function through the Erk pathway [34].

*Cyclin D1 *and *c-myc *are key regulatory genes in the control of cell cycle and cell proliferation, and thus are the best-known candidates among the LEF-1 regulated genes [35,36]. Over-expression of *cyclin D1 *ranged from 5.6% to 54% of HCCs and was associated with advanced clinicopathological stage [30]. Up-regulation of *c-myc *gene was reported by Kawate et al in 33% of HCCs by differential PCR analysis [37]. However, to date, the roles of *cyclin D1 *and *c-myc *in HCCs are still not well defined. In this study, expression of *cyclin D1 *and *c-myc *was markedly increased in HCC tissues, compared with normal liver tissues but the expression levels of these two genes were higher in peritumor cells than that of tumor cells. This could partly be attributed to the over-expression of 38 kDa dominant negative LEF-1 isoform in tumor cells. Up-regulation of 38 kDa dominant negative isoform of LEF-1 in tumor cells could suppress rather than activate the *Wnt *pathway. Therefore the downstream target genes, *cyclin D1 *and *c-myc*, were induced at a lower level in the tumor cells, compared to that of peritumor cells. However the complexity of *cyclin D1 *and *c-myc *in HBV-associated HCC tissues should be considered.

## Conclusion

Taken together, as there was higher expression of HBsAg in peritumor cells and higher up-regulation of LEF-1 in the cytoplasm of cells, as well as higher up-regulation of *cyclin D1 *and *c-my*, it is predicted that HBsAg exerted pronounced effects on LEF-1 and its downstream genes in hepatocytes, resulting in more active cell proliferation, which could promote or enhance malignant transformation of hepatocytes by other viral or cellular mechanisms. It is postulated that HBsAg interacted with liver cells only at the pre-malignant stage, and thus plays the role of an initiator during the process of HCC development.

## Competing interests

The authors declare that they have no competing interests.

## Authors' contributions

XT carried out molecular studies, collected and analyzed the data, performed the statistical analysis and drafted the manuscript. JL carried out IHC studies. MZM and CZ carried out part of real-time PCR studies. WDF collected the samples and participated in the design of the study. YMW designed the concept of this study and approved the final manuscript. All authors read and approved the final manuscript.
